# Correction: Validation of the Arabic Levels of Emotional Awareness Scale (LEAS-Arb)

**DOI:** 10.1371/journal.pone.0350819

**Published:** 2026-06-02

**Authors:** Maimounah Hebi, Johanna Czamanski-Cohen, Karen L. Weihs, Richard D. Lane

In Fig 2, the example in this figure is not correctly presented for the level 4 and level 5 scores. Please see the correct Fig 2 here.

**Fig 2:**
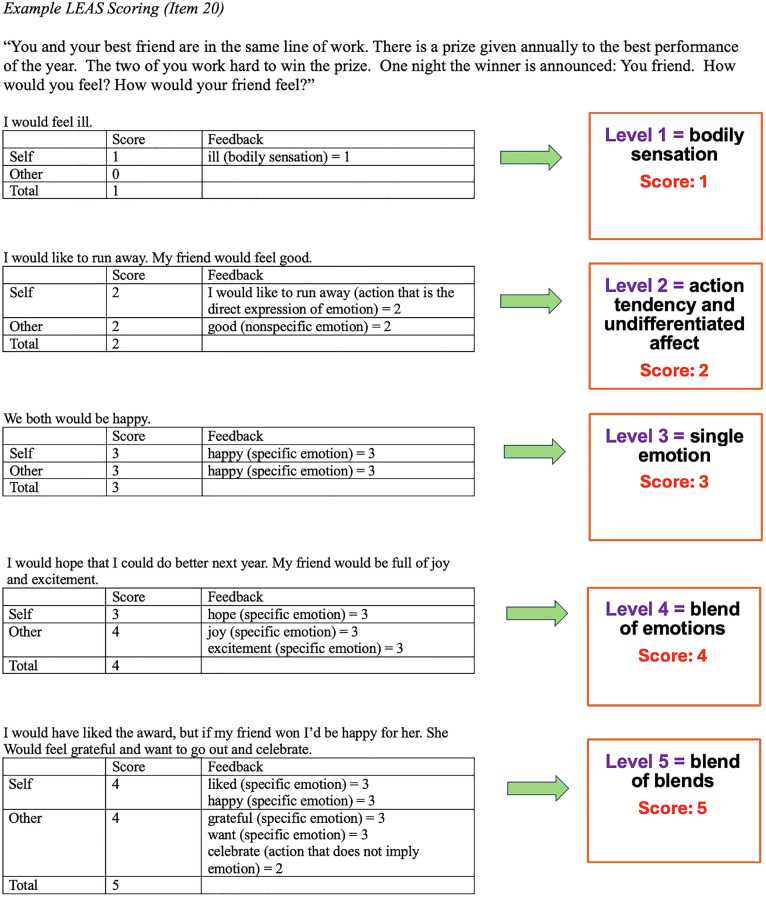
Example LEAS Scoring (Item 20).
