# Supplementary material for: Validation of the Arabic Levels of Emotional Awareness Scale (LEAS-Arb)
Source: PLoS One. 2026 Apr 9;21(4):e0332559. doi: 10.1371/journal.pone.0332559 (PMC13065075; doi:10.1371/journal.pone.0332559)
Supplement: Attachment — Submitted filename: ResponseLetter.docx [file pone.0332559.s002.docx]

Dear Editor and Reviewers,

We would like to thank the Editor and Reviewers for their careful evaluation of our manuscript and for the constructive and helpful comments. We have revised the manuscript accordingly and believe that these changes have substantially improved its clarity, rigor, and presentation. Below, we provide a point-by-point response to all comments. All changes in the manuscript are indicated in the revised version.

1. We revised the manuscript and associated files to ensure full compliance with PLOS ONE style and file-naming requirements, following the journal’s formatting sample guidelines.
2. We completed the PLOS ONE Inclusivity in Global Research questionnaire and have uploaded it as Supporting Information with the revised submission.
3. We updated the Competing Interests statement in the cover letter to explicitly confirm that this disclosed interest does not alter our adherence to PLOS ONE policies on sharing data and materials. No additional restrictions apply beyond those described in the Data Availability Statement.
4. We revised the Data Availability Statement to clarify that public deposition of the full dataset would breach compliance with the protocol approved by the University of Haifa Ethics Committee due to the sensitive nature of the human participant data. Accordingly, we request an ethics-based exemption and specify that deidentified data are available through a controlled access process, as detailed in the revised statement.

**Reviewer #1, Comment (a):**
**The abstract should include a mention of the convergent validity of the instrument with respect to alexithymia and positive mood. The adaptation cannot be defended solely based on the internal consistency indices.**

We revised the abstract to explicitly report evidence of convergent validity, including theoretically consistent associations with alexithymia and positive mood, in addition to internal consistency indices.

**Reviewer #1, Comment (b):**
**The discussion should critically analyse the limited evidence of convergent validity with respect to alexithymia, given that significant correlations have only been found with the Externally Oriented Thinking component.**

We expanded the Discussion section to critically address the limited convergent validity observed with alexithymia. Specifically, we clarified that the selective association with the Externally Oriented Thinking subscale replicates prior large-scale findings and discussed theoretical and methodological explanations for this pattern, including differences between self-report and performance-based measures.

**Reviewer #1, Comment (c):**
**Add to the limitations the fact that the factorial structure has not been explored. It could be added that this has been postponed for a later study once a larger and sufficient sample size has been achieved.**

We added this point to the Limitations section, noting that the factorial structure of the LEAS-Arb was not examined due to sample size considerations and that factor analytic procedures are planned for future research once a larger and more statistically adequate sample is available.

**Reviewer #2, Comment:**
**The introduction needs to elaborate on the significance of the LEAS and its application in other countries.**

We expanded the Introduction to elaborate on the significance of the LEAS and its international applications. Specifically, we highlighted evidence that emotional awareness is a trainable skill, the suitability of the LEAS for assessing change following intervention, and the importance of administering the instrument in respondents’ native language, with reference to prior language-specific validations in other countries.

**Reviewer #2, Comment:**
**When giving examples of LEAS scoring, please verify the case of the full score of 5 points. Only when both the self and other dimensions are scored 4 points can the total score be counted as 5 points.**
We clarified the LEAS scoring procedure in the Methods section to specify that a total score of 5 is assigned only when both self and other responses receive a score of 4 and contain distinct, non-redundant emotional content; otherwise, the total score is 4. This clarification appears in the paragraph describing LEAS scoring, immediately preceding the reference to Figure 2.
